# Monitoring chest compression quality during cardiopulmonary resuscitation: Proof-of-concept of a single accelerometer-based feedback algorithm

**DOI:** 10.1371/journal.pone.0192810

**Published:** 2018-02-14

**Authors:** Digna María González-Otero, Jesus María Ruiz, Sofía Ruiz de Gauna, Jose Julio Gutiérrez, Mohamud Daya, James Knox Russell, Izaskun Azcarate, Mikel Leturiondo

**Affiliations:** 1 Department of Communications Engineering, University of the Basque Country (UPV/EHU), Bilbao, Bizkaia, Spain; 2 Department of Emergency Medicine, Oregon Health & Science University (OHSU), Portland, Oregon, United States of America; Vanderbilt University Medical Center, UNITED STATES

## Abstract

**Background:**

The use of real-time feedback systems to guide rescuers during cardiopulmonary resuscitation (CPR) significantly contributes to improve adherence to published resuscitation guidelines. Recently, we designed a novel method for computing depth and rate of chest compressions relying solely on the spectral analysis of chest acceleration. That method was extensively tested in a simulated manikin scenario. The purpose of this study is to report the results of this method as tested in human out-of-hospital cardiac arrest (OHCA) cases.

**Materials and methods:**

The algorithm was evaluated retrospectively with seventy five OHCA episodes recorded by monitor-defibrillators equipped with a CPR feedback device. The acceleration signal and the compression signal computed by the CPR feedback device were stored in each episode. The algorithm was continuously applied to the acceleration signals. The depth and rate values estimated every 2-s from the acceleration data were compared to the reference values obtained from the compression signal. The performance of the algorithm was assesed in terms of the sensitivity and positive predictive value (PPV) for detecting compressions and in terms of its accuracy through the analysis of measurement error.

**Results:**

The algorithm reported a global sensitivity and PPV of 99.98% and 99.79%, respectively. The median (P_75_) unsigned error in depth and rate was 0.9 (1.7) mm and 1.0 (1.7) cpm, respectively. In 95% of the analyzed 2-s windows the error was below 3.5 mm and 3.1 cpm, respectively.

**Conclusions:**

The CPR feedback algorithm proved to be reliable and accurate when tested retrospectively with human OHCA episodes. A new CPR feedback device based on this algorithm could be helpful in the resuscitation field.

## Introduction

According to current resuscitation guidelines, high quality cardiopulmonary resuscitation (CPR) is essential to increasing survival from out-of-hospital cardiac arrest (OHCA) [1, 2]. During CPR, the early and persistent application of chest compressions and ventilations to the patient artificially maintains a minimal flow of oxygenated blood. This delays brain damage and generates myocardial blood flow, essential to the restoration of a perfusing rhythm. Based mainly on observational studies [3–5], published guidelines recommend compression depths between 50 and 60 mm, at a rate between 100 and 120 compressions per minute (cpm), allowing complete chest recoil between compressions and minimising interruptions.

Unfortunately, delivering adequate chest compressions is difficult both for laypeople [6] and well trained rescuers [7, 8]. Pauses between compressions are very frequent, and compressions are often too fast and/or too shallow. Consequently, monitoring CPR using feedback devices to guide rescuers during resuscitation attempts has been increasingly investigated in recent years. These devices measure in real-time core parameters such as compression depth and rate, helping the rescuer to correct the technique if necessary. To date, there is strong evidence that feedback improves chest compression quality in training and in real practice [9–12].

Since the appearance of the first devices based on force sensors, technology has evolved towards systems based on accelerometers, which sense the chest acceleration during compressions and calculate the instantaneous chest displacement by double integration of the chest acceleration. This computation is challenging due to cumulative integration errors which must be compensated to obtain a reliable compression displacement [13–15]. For this purpose, commercial accelerometer-based feedback systems include additional force/pressure sensors or other reference signals to fix boundary conditions for the integration process [12, 13]. Gonzalez-Otero et al. recently published a novel algorithm for computing chest compression depth and rate based exclusively on the spectral analysis of the acceleration [16]. This algorithm required no additional sensor or reference signal, and proved to be accurate after extensive testing in laboratory conditions using a sensorized resuscitation manikin.

Acceleration patterns observed when compressions are provided on a manikin in a controlled scenario may strongly differ from those observed during real resuscitation episodes in humans. First, a human chest has a non-linear stiffness (force-depth relationship) which varies among individuals and changes during the resuscitation attempt [17]. This may result in different acceleration patterns compared to the simulated spring-based manikin model, which mimics a perfectly elastic chest. Second, several rescuers are usually involved in each intervention, and as a result different acceleration patterns are likely to be observed depending on the technique used to apply and remove the force from the chest. Third, resuscitation attempts can last more than 30-40 minutes [18], and rescuer fatigue can result in more irregular acceleration patterns during compression series. This could be particularly apparent during the latter half of the resuscitation effort. Additionally, a decline in chest compression quality has been observed in the minutes prior to scene departures [19]. These differences suggest that performance results derived from a simulated study may not be directly extrapolated to real practice.

In this study, we present the proof of concept of the spectral analysis CPR feedback method based on acceleration [16]. The method was applied retrospectively to the chest acceleration data recorded during human OHCA episodes. We analyzed the reliability and accuracy of the method in the computation of chest compression depth and rate.

## Materials and methods

### Data collection

Data were extracted from a large database of 623 OHCA episodes collected between 2006 and 2009 by Tualatin Valley Fire & Rescue (TVF&R), an advanced life support first response emergency medical service system serving nine incorporated cities in Oregon, USA. Episodes were recorded with Heartstart MRx monitor-defibrillators (Philips Medical Systems, Andover, MA, USA) and collected as part of the Resuscitation Outcomes Consortium (ROC) Epidemiological Cardiac Arrest Registry. The data collection for the ROC Epistry was approved by the Oregon Health & Science University (OHSU) Institutional Review Board (ID: IRB00001736). No clinical data was available for this study.

Defibrillators were equipped with real-time CPR feedback technology (Q-CPR, Laerdal Medical, Norway), based on chest acceleration and compression force sensing. The acceleration signal was acquired by the accelerometer fitted in the Q-CPR device with a sampling rate of 250 Hz. The compression signal was calculated from the acceleration and the force signals using a proprietary algorithm. Signals were stored in Matlab (Mathworks, MA, USA) format with a sampling frequency of 250 Hz and a 16-bit resolution.

### Data annotation

We included in our study those episodes containing concurrent chest acceleration and compression signals with a minimum duration of 20 min, more than 1500 compressions and with a minimum average depth of 30 mm. These criteria allowed the inclusion of episodes in which Q-CPR was used during a significant part of the episode and that were long enough to present representative variations of the acceleration patterns associated to out-of-hospital CPR.

**Fig 1:**
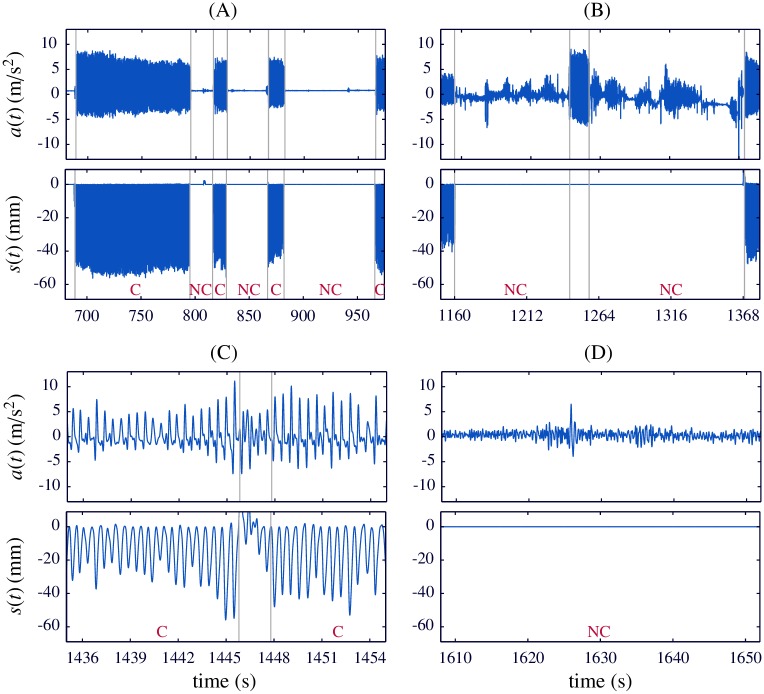
Interval selection. Graphical examples showing selected and discarded intervals in the episodes. (A) Selected intervals of compressions (C) and no-compressions (NC). (B) Q-CPR compression signal is not available in the presence of chest compressions. (C) Interval with non-consistent computation of compression signal. (D) Noisy acceleration during a compression pause.

We used a Matlab custom-made program for visually inspecting the episodes. We identified the onset and offset of each chest compression series using the Q-CPR compression signal. Thus, compression and no-compression intervals were annotated for reference and included in the analysis. Fig 1(A) shows an example of the included intervals. Note that compression signal is depicted with negative values meaning downward chest displacement.

We discarded intervals in which either the acceleration or the compression signals were not reliable. Specifically, we excluded:

- Intervals with discontinuous recordings of acceleration and/or compression signals. Fig 1(B) shows an example: compressions are being provided, as can be observed in the acceleration signal (top). However, the compression signal (bottom) is a flat line, providing no reference for this compression interval.
- Intervals with computing errors in the compression signal. In Fig 1(C), the compression signal enclosed between gray lines shows non-consistent positive values.
- Intervals with very noisy acceleration, as illustrated in Fig 1(D). Spiky or random acceleration patterns, non-consistent with acceleration waveform during chest compressions, were also discarded. All intervals of this type corresponded to hands-off periods, i.e. with no chest compressions being administered to the patient. These were isolated moments in which the Q-CPR device was apparently removed from the patient chest, so the rescuer was not expecting feedback.

### Feedback method

The core algorithm for computing chest compression depth and rate exclusively from the spectral analysis of the acceleration was described previously [16]. The principle of this algorithm relies on the quasi-periodicity of the chest acceleration and displacement during short intervals of chest compressions. For every 2-s acceleration interval, henceforth referred to as 2-s window, the algorithm provides a value of average depth and a value of average rate provided that compression activity is detected.

**Fig 2:**
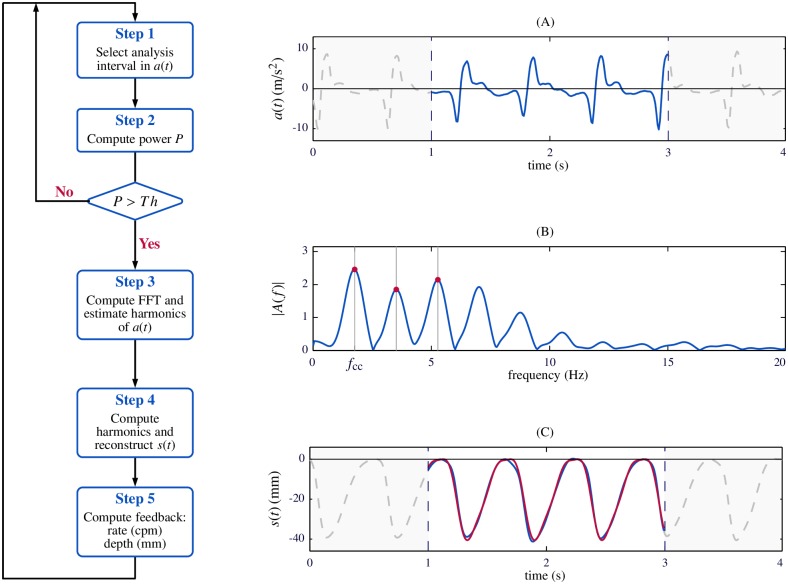
Flow diagram of the feedback algorithm. Step 1: selection of the 2-s window. Step 2: detection of compression activity from the power *P* of the 2-s window (A). Step 3: FFT computation for estimation of acceleration harmonics (B). Step 4: reconstruction of compression signal (C). Reconstructed signal is depicted in red, and the reference signal in blue. Step 5: calculation of feedback rate and depth values.

The flow diagram of the feedback algorithm during continuous analysis of the acceleration signal is depicted in Fig 2. In step 1, a new 2-s window is selected in the acceleration signal (panel A). In step 2, presence or absence of chest compressions is tested. For this purpose, the power *P* of the 2-s window is computed and if *P* exceeds a fixed threshold then the window is classified as *compression window*. If this occurs, the algorithm continues to step 3. Otherwise, the 2-s window is classified as *no-compression window*. In step 3, the Fast Fourier Transform (FFT) of the 2-s window is computed, and the fundamental frequency and the first three harmonics are estimated in the spectral domain. Fig 2, panel B, shows the modulus of the FFT, where the fundamental frequency, *f*_*cc*_, and the harmonics are annotated with vertical lines. These data are then used in step 4 to compute the Fourier series representation of the displacement signal, a periodic signal which corresponds to the average compression signal in the observed window (Fig 2, panel C). Feedback values are finally calculated from this signal (step 5): compression rate is the fundamental frequency of the acceleration measured in the FFT expressed in cpm; compression depth is the peak-to-peak value of the average compression signal in mm. For the example in Fig 2, computed rate and depth was 105.5 cpm and 40.6 mm, respectively.

Our algorithm provides quasi real-time feedback, as it computes the average depth and rate exerted by the rescuer in the last 2 seconds. Assuming non-overlapping analysis windows, the feedback refreshing interval would be 2 s. This feedback timing provides a balance between quickly detecting changes in CPR performance and not supplying excessive information that could stress the rescuer. The algorithm is simple and has a low computational cost. In a processor typically used by monitor-defibrillators, the processing of the 2-s acceleration window and the computation of feedback values require less than 0.1 s.

### Data analysis and performance evaluation

The algorithm was applied to the acceleration signal of each episode, divided into non-overlapping 2-s windows. The reliability of the compression detector was assessed in terms of sensitivity and positive predictive value (PPV) as figures of merit. Sensitivity was defined as the proportion of correctly detected *compression windows* among all 2-s windows in which presence of chest compressions was annotated. PPV value was the proportion of correctly detected *compression windows* among all the positive detections.

We evaluated the accuracy of the feedback algorithm as follows: for every 2-s window correctly detected as *compression window*, we compared the depth and rate feedback values provided by the algorithm with the gold standard (GS) values obtained from the chest compression signal provided by the Q-CPR system.

GS depth was computed by averaging the depth of the compression events included in the analyzed 2-s window. Similarly, GS rate was computed as the inverse of the averaged time interval between consecutive compression events in the analyzed 2-s window. The figure of merit for accuracy was the measurement error in depth and in rate, computed as the difference between the estimate and the GS value.

As data did not pass the Lilliefors normality test, median and percentiles were reported. We studied the measurement error globally (for the whole dataset) using histograms and compared them to the gold standard using Bland-Altman plots. We also characterized the unsigned measurement error globally and per episode.

## Results

From the initial 623 OHCA episodes, only 75 fulfilled all the inclusion criteria. Concurrent acceleration and chest compression signals were available in 558 episodes. From these, 262 had a duration superior to 20 minutes but only 79 had also more than 1500 compressions. From these, 75 had a mean compression depth higher than 30 mm.

According to the annotation criteria, the total discarded time was 6.8%. A total of 57142 2-s acceleration windows were analyzed, 41912 (73%) were annotated as *compression window*. The median (P_25_-P_75_) number of annotated *compression window* and *no-compression window* per record was 531 (461-640) and 175 (106-258), respectively.

**Fig 3:**
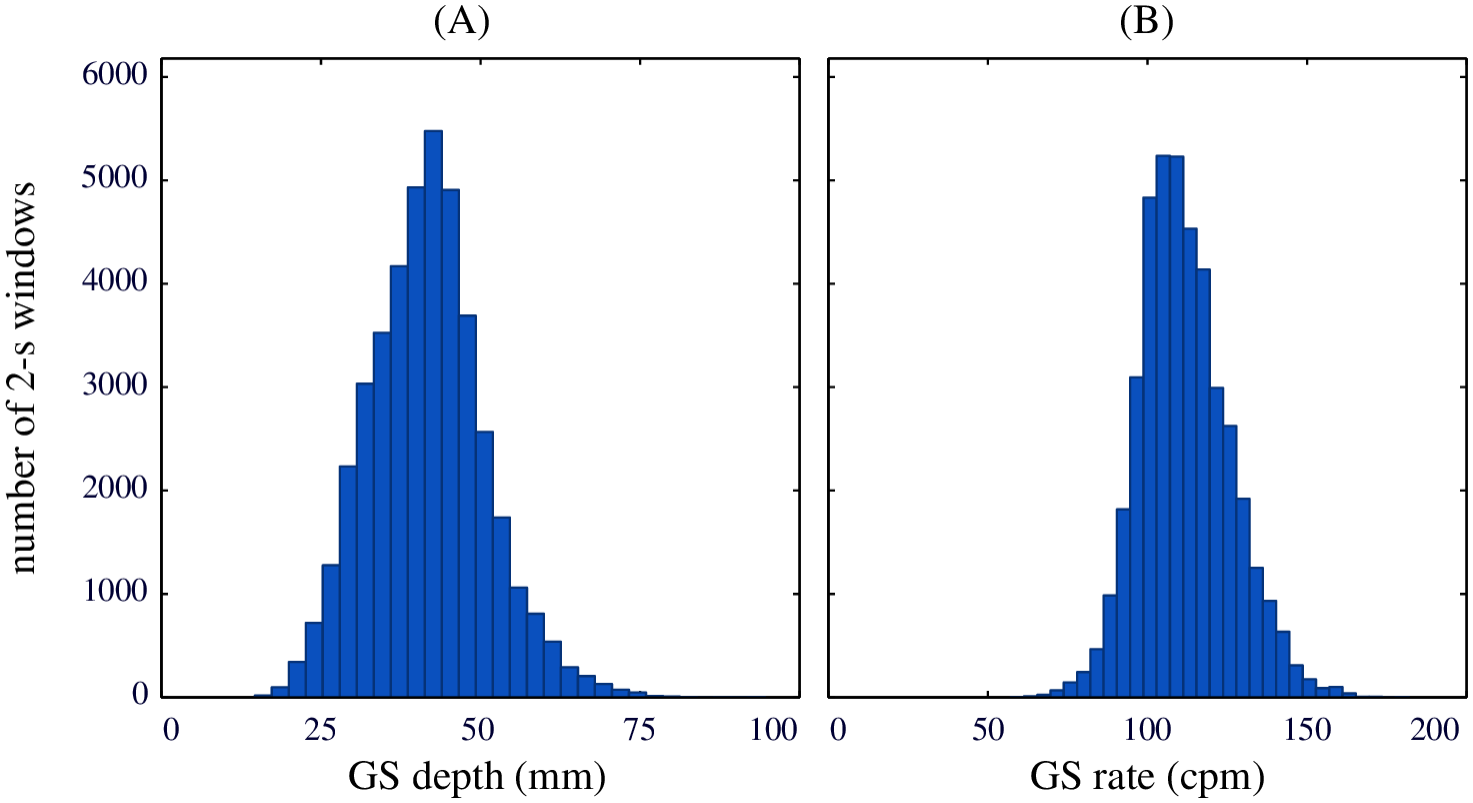
Distribution of the GS values. Distribution of the GS compression values for depth (left) and rate (right) in the analyzed 2-s windows.

Fig 3 shows the histograms of the GS values for depth (panel A) and rate (panel B). Compressions were provided with a median depth of 41.6 (35.4-47.0) mm, in a range from 14.6 to 94.6 mm. Compression rate was 110.3 (101.9-120.2) cpm, in a range from 57.1 to 181.9 cpm. Target depth and rate recommended by the 2005 American Heart Association guidelines (in force during the period during which the episodes were gathered) were 38-50 mm and approximately 100 cpm, respectively [20].

Table 1 presents the confusion matrix used for evaluating the compression detector (step 2 in the feedback algorithm). Global sensitivity and PPV were 99.98% and 99.79%, respectively. Only ten of the 41912 *compression windows* (0.02%) were not detected (FN in the confusion matrix). On the other hand, 90 of the 15230 *non-compression windows* (0.59%) resulted in FP events.

**Table 1:** Confusion matrix for the compression detector.

	Comp. window	No comp. window	Total
**Comp. detected**	TP: 41902	FP: 90	41992
**No comp. detected**	FN: 10	TN: 15140	15150

TP (True Positive), FP (False Positive), FN (False Negative), TN (True Negative).

**Fig 4:**
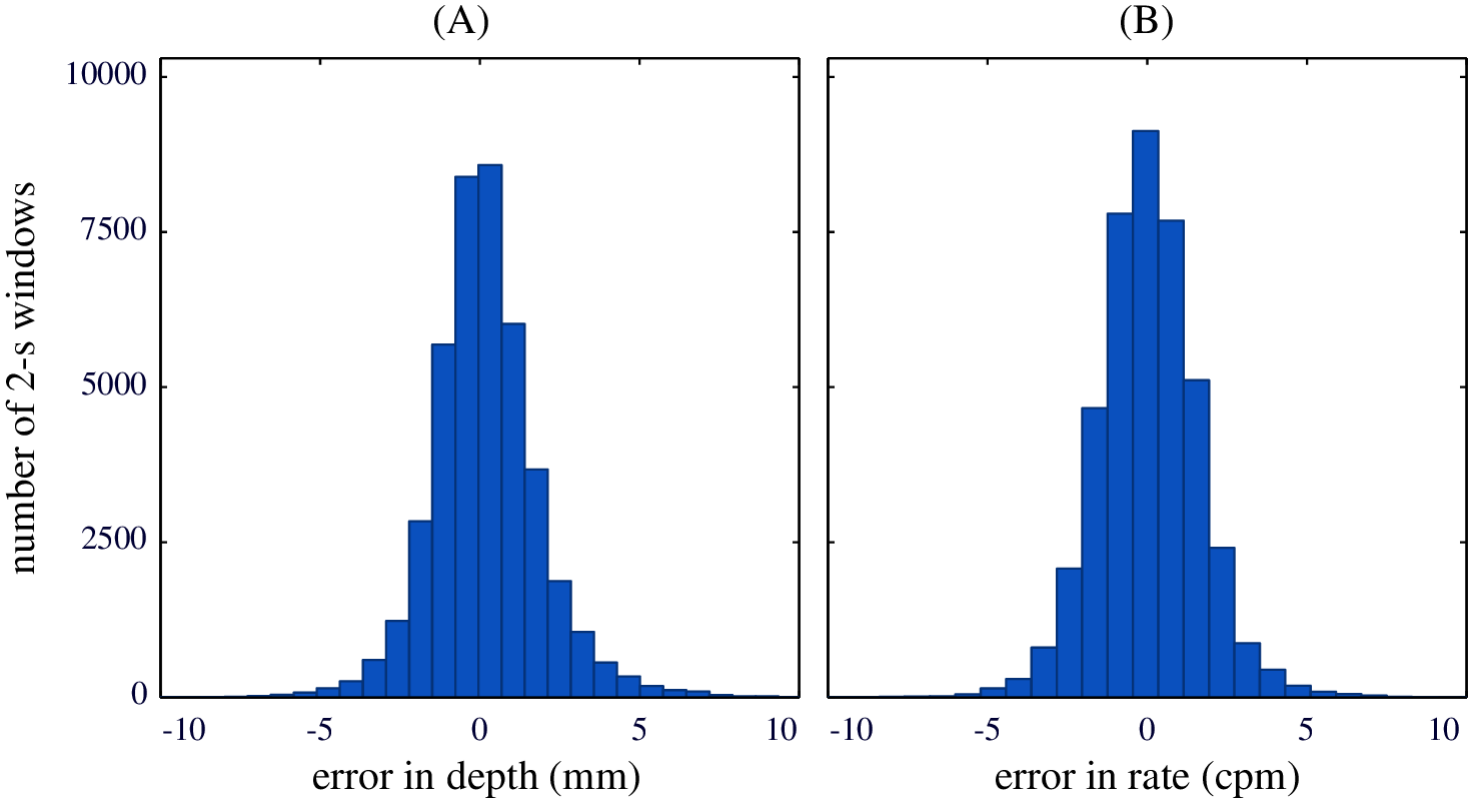
Distributions of the measurement error. Distribution of the error values for depth (left) and rate (right) in the analyzed 2-s windows.

**Fig 5:**
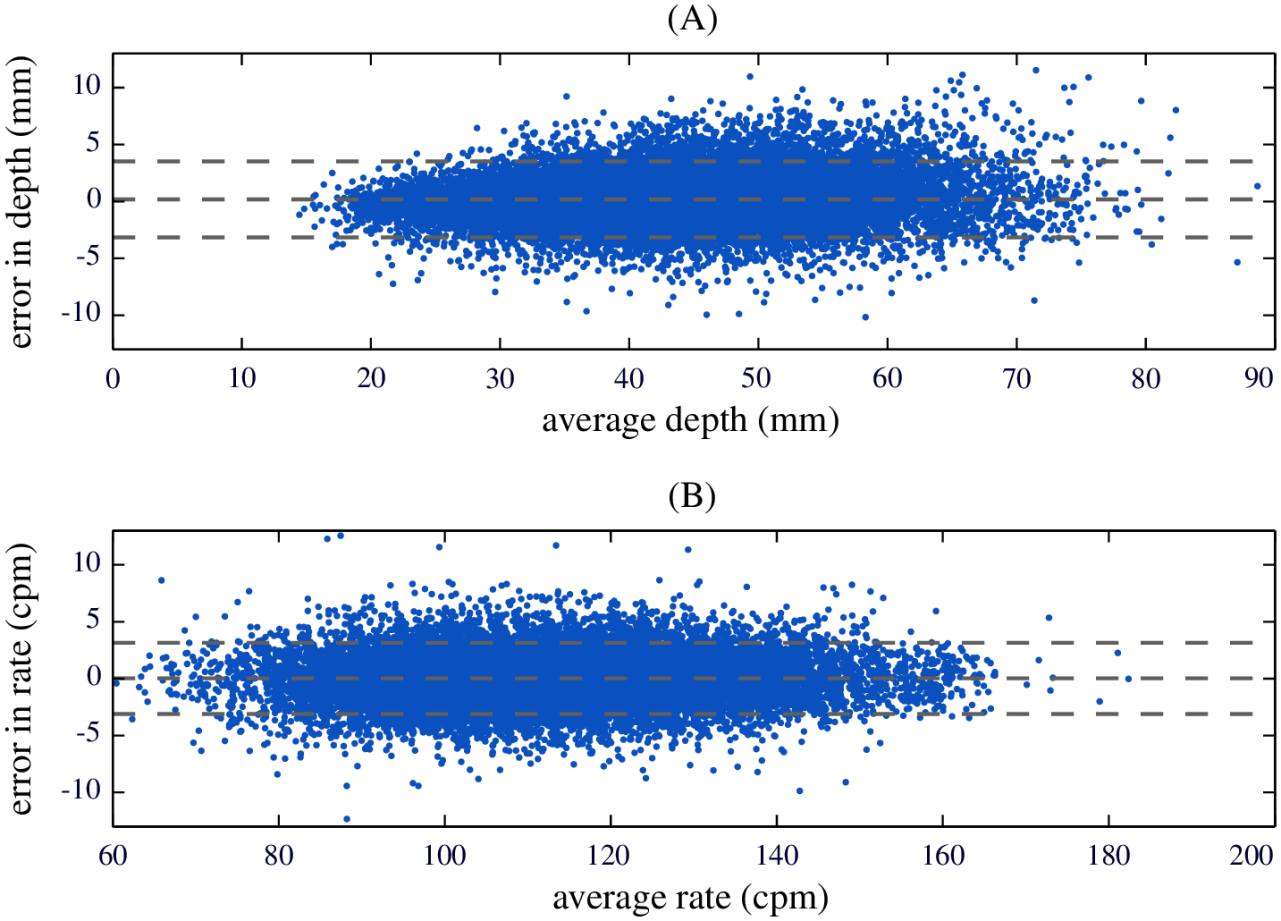
Measurement error against average of estimate and GS value. Compression depth (A), compression rate (B). Mean and 95% levels of agreement are depicted with dashed lines.

Fig 4 depicts the distributions of the measurement error in depth (panel A) and in rate (panel B). Fig 5 shows two Bland-Altman plots depicting the measurement error as a function of the average of estimate and GS value. Panel A refers to compression depth measurement and panel B to compression rate. Mean and 95% limits of agreement, depicted with dashed lines in the figure, were 0.18 (-3.16, 3.52) mm and 0.02 (-3.10, 3.14) cpm for depth and rate, respectively.

Table 2 shows the median value and percentiles of the unsigned (absolute) measurement error for the whole population. Median unsigned error in depth and rate was 0.9 mm and 1.0 cpm, respectively. In 95% of the analyzed 2-s windows the error was below 3.5 mm and 3.1 cpm, respectively.

**Table 2:** Unsigned error in the estimation of compression depth and rate.

	Unsigned error					
Parameter	Median	P_75_	P_90_	P_95_	P_99_	Range
**Depth (mm)**	0.9	1.7	2.7	3.5	5.6	0-11.5
**Rate (cpm)**	1.0	1.7	2.5	3.1	4.8	0-12.3

Regarding the statistics per episode, the median unsigned error per episode in depth and rate varied from 0.5 to 1.9 mm, and from 0.8 to 1.4 cpm, respectively. Similarly, the 75th percentile per episode varied from 0.9 mm to 3.3 mm, and from 1.3 cpm to 2.5 cpm.

### Performance examples

**Fig 6:**
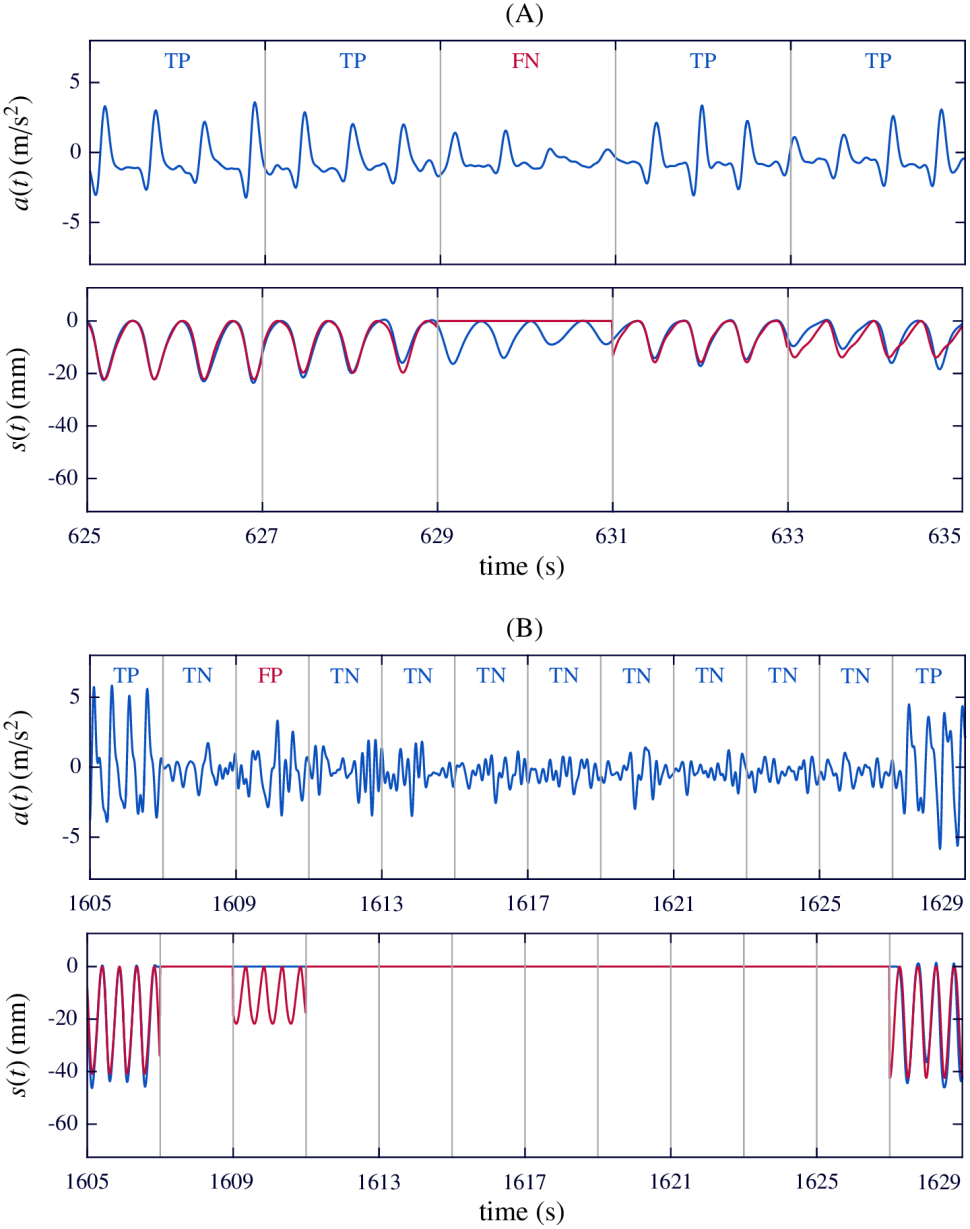
Examples of algorithm performance. (A) Isolated false negative. Central 2-s window shows a weak acceleration and compressions were not detected. Q-CPR compression signal (blue) indicated a very low depth in this interval. Computed compression signal (red) was reconstructed as a flat line. (B) Isolated false positive. Noisy acceleration during a hands-off interval, resulting in false detection of compression activity.

Fig 6 shows two examples of algorithm performance. Example (A) depicts an isolated false negative event in an interval with low compression depth (below 20 mm). In the 2-s window marked as FN, the power of the acceleration was below the fixed threshold, so it was classified as *no-compression window*. The algorithm provided no feedback, and the reconstructed compression signal was a flat line (depicted in red in the bottom panel, with the reference overlapped in blue). Note that the four compression events in the analyzed window had a depth of around 10 mm. This very low depth was rarely found in the episodes. Example (B) shows an isolated false positive event. The acceleration was very noisy and compression activity was detected in the 2-s window marked as FP. In that interval, no chest compressions were delivered, so the rescuer was not expecting feedback.

**Fig 7:**
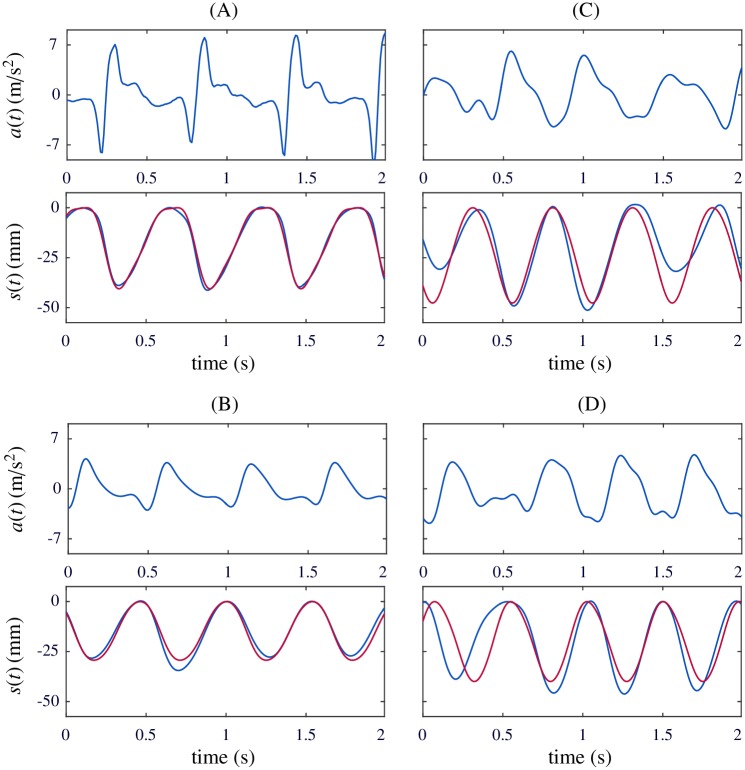
Examples of algorithm accuracy. Good accuracy: (A) Depth error was 0.5 mm and rate error was -0.7 cpm. (B) Depth error was -0.1 mm and rate error was 0.2 cpm. Poor accuracy due to lack of acceleration periodicity: (C) Depth error was 6.9 mm and rate error was 1.9 cpm. (D) Depth error was -3.9 mm and rate error was 6.5 cpm.

Fig 7 shows four examples of algorithm accuracy. For each example, the acceleration signal is depicted above the estimated compression signal (in red, overlapped to the reference signal in blue). Example (A) shows a very regular compression displacement whereas in example (B) reference compression signal varies along the 2-s interval. Examples (C) and (D) show important computation errors caused by non-periodic acceleration waveforms. This might be expected during the latter half of a resuscitation effort as rescuers fatigue may complicate a rhythmic compression pattern.

**Fig 8:**
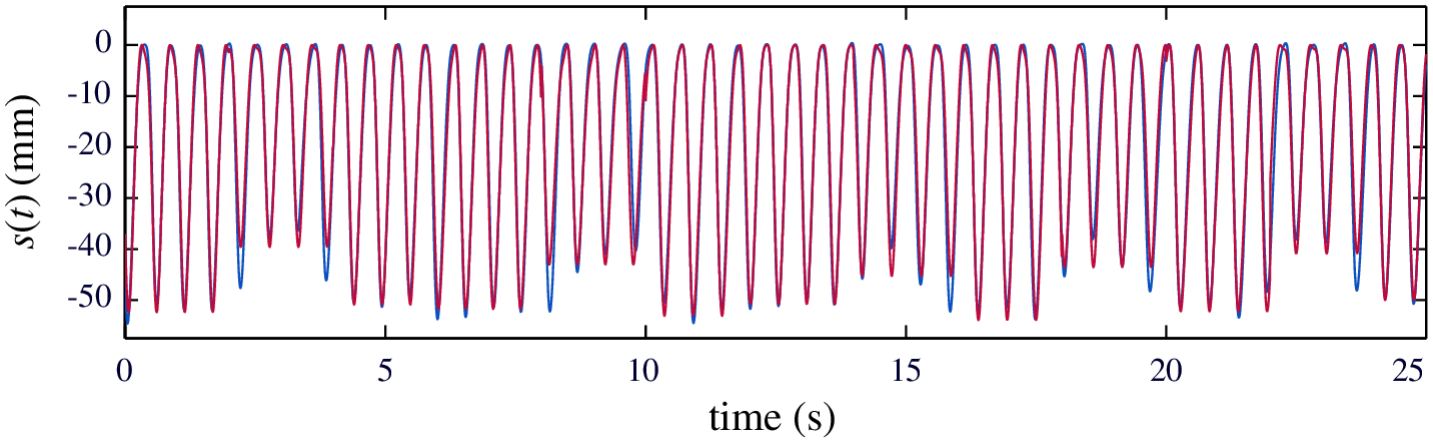
Reconstructed compression signal. Computed compression signal is depicted in red with the overlapped GS compression signal computed by the Q-CPR technology (in blue).

In addition to real-time feedback, the algorithm allows the complete reconstruction of the compression depth signal for the entire episode by the concatenation of the consecutive 2-s periodic compression segments. This provides a reliable estimate of the chest displacement along the entire episode, and properly accounts for its local variations. This is shown in Fig 8, which depicts the computed compression signal (in red) and the reference signal (in blue). Availability of the entire compression signal per episode is useful for debriefing process by the emergency crews.

## Discussion

Monitoring CPR performance by rescuers at the scene of cardiac arrest has become essential in the science of resuscitation. Development of accurate and widely available CPR feedback devices is a key component to improve performance. Feedback relying solely on accelerometry sensing could be simple to implement in defibrillators. In particular, incorporating the real-time feedback functionality in automated external defibrillators could contribute to enhancing bystander chest compressions quality.

This study is the proof-of-concept of a novel chest compression feedback algorithm capable of providing real-time help to the rescuers during CPR using only accelerometers. The algorithm detects the presence of chest compressions in the acceleration and, when they are present, measures the mean depth and rate of the compressions given by the rescuer every 2-s. The algorithm can perform in real-time (feedback on the compression quality in the last 2-s) and also allows the reconstruction of the entire displacement signal per episode, useful for debriefing purposes.

Results proved the good reliability of the feedback algorithm in the detection of chest compressions. Only 0.02% of the 2-s windows with chest compressions were not detected. This represents a negligible time when the rescuer would receive no feedback during chest compressions. In general, this corresponded to isolated intervals with very shallow chest compressions rarely found in advanced life support CPR. During the basic life support sequence of 2 min 30:2 CPR, the defibrillator could advise the rescuer to compress according to guidelines if no compressions are detected. On the other hand, in only 0.59% of the windows, compressions were falsely detected. This would have no negative impact on CPR, as the rescuer is not actually delivering chest compressions.

The algorithm also performed very accurately, globally and per episode, according to the analysis of the measurement error. Irregular non-periodic acceleration intervals clearly affecting the accuracy of depth and rate were mainly observed in the latter half of the resuscitation effort, and might be attributable to fatigue or patient movement during chest compressions prior to transportation.

Our method uses exclusively the chest acceleration signal to provide feedback on compression depth and rate and makes the continuous compression depth signal available during the entire episode. In contrast, Q-CPR and most commercial accelerometer-based devices require an additional sensor (force/pressure sensor) or other reference signal for an accurate computation of the compression depth signal. This increases processing complexity. Protection of force/pressure sensors makes device rigid and bulkier, and after prolonged CPR this could result in damage to the patient and cause rescuer’s discomfort [12, 21]. In contrast, accelerometers could be inbuilt in a flexible encasement. This may lead to simpler, cheaper and flexible devices with a low computational cost, but with a comparable measurement accuracy.

The major drawback of using acceleration only is the impossibility to detect chest recoil between compressions. This limitation was discussed in our previous publications [14, 16]. Q-CPR technology relies on the force (additional sensor) for detecting rescuer’s leaning. However, human chest stiffness is non-linear, varies strongly among patients and with time along the resuscitation episode. Consequently, measuring leaning with force gives no accurate information about the recoil value (that is, how many millimeters is the chest being still compressed when it should be completely released). In consequence, force can only be used as an indirect binary indicator of leaning.

Another limitation of CPR feedback devices based on a single accelerometer is that they overestimate chest compression depth when the patient is laying on a soft surface, such as a mattress [22, 23]. The accelerometer senses chest displacement, which in this case corresponds to the sum of chest compression and mattress compression. A possible solution to this problem would be to use two accelerometers. We explored the accuracy of our algorithm when using two accelerometers to compensate for mattress displacement elsewhere [24]. The data analyzed in this study were collected when CPR was provided with the patient laying on the floor, so this limitation does not affect the results.

Current CPR guidelines are dated 2015, and compression depth and rate standards have changed since 2005. Compression depth target has increased from 38-50 mm to 50-60 mm, based on observational data which suggested improved outcomes in association with deeper compressions [25]. Consequently, OHCA episodes used in our study showed a lower median compression depth (41.6 mm) compared with current recommendation. Compression rate target has also slightly changed from approximately 100 cpm to 100-120 cpm. Despite that, median compression rate in our episodes was 110.3 cpm, consistent with current rate target.

The main limitation of this study is the absence of a true gold standard to test the reliability and accuracy of our novel feedback algorithm. We relied on the compression and rate values estimated by the commercial Q-CPR device. Despite this limitation, we think it is a reasonable surrogate measure for performance comparison using real OHCA episodes.

## Conclusion

The feedback algorithm discussed here proved to be reliable and accurate when tested retrospectively with human cardiac arrest episodes. Consequently, a new CPR feedback device based on this algorithm could be helpful in the resuscitation field, both in training and in clinical scenarios.

## Supporting information

S1 File Results corresponding to the computation of chest compression depth and rate. Estimated vs. Gold Standard values for the analyzed 2-s windows per episode.
(XLS)

S2 File Matlab code. Code used to visualize the results (signals and values) in Matlab.
(ZIP)

## References

[1] Perkins GD, Handley AJ, Koster RW, Castrén M, Smyth MA, Olasveengen T, et al European Resuscitation Council Guidelines for Resuscitation 2015: Section 2. Adult basic life support and automated external defibrillation. Resuscitation. 2015;95:81–99. doi: 10.1016/j.resuscitation.2015.07.015 26477420 10.1016/j.resuscitation.2015.07.015

[2] Kleinman ME, Brennan EE, Goldberger ZD, Swor RA, Terry M, Bobrow BJ, et al Part 5: Adult Basic Life Support and Cardiopulmonary Resuscitation Quality. 2015 American Heart Association Guidelines Update for Cardiopulmonary Resuscitation and Emergency Cardiovascular Care. Circulation. 2015;132(18 suppl 2):S414–S435. doi: 10.1161/CIR.0000000000000259 26472993 10.1161/CIR.0000000000000259

[3] Idris AH, Guffey D, Pepe PE, Brown SP, Brooks SC, Callaway CW, et al Chest compression rates and survival following out-of-hospital cardiac arrest. Critical care medicine. 2015;43(4):840–848. doi: 10.1097/CCM.0000000000000824 25565457 10.1097/CCM.0000000000000824

[4] Stiell IG, Brown SP, Nichol G, Cheskes S, Vaillancourt C, Callaway CW, et al. What is the optimal chest compression depth during out-of-hospital cardiac arrest resuscitation of adult patients? Circulation. 2014; p. CIRCULATIONAHA–114. 10.1161/CIRCULATIONAHA.114.008671 25252721

[5] Christenson J, Andrusiek D, Everson-Stewart S, Kudenchuk P, Hostler D, Powell J, et al Chest compression fraction determines survival in patients with out-of-hospital ventricular fibrillation. Circulation. 2009;120(13):1241–1247. doi: 10.1161/CIRCULATIONAHA.109.852202 19752324 10.1161/CIRCULATIONAHA.109.852202 PMC2795631

[6] Gallagher EJ, Lombardi G, Gennis P. Effectiveness of bystander cardiopulmonary resuscitation and survival following out-of-hospital cardiac arrest. JAMA: the Journal of the American Medical Association. 1995;274(24):1922–1925. doi: 10.1001/jama.1995.03530240032036 8568985

[7] Abella BS, Alvarado JP, Myklebust H, Edelson DP, Barry A, O’Hearn N, et al Quality of cardiopulmonary resuscitation during in-hospital cardiac arrest. JAMA: the Journal of the American Medical Association. 2005;293(3):305–310. doi: 10.1001/jama.293.3.305 15657323 10.1001/jama.293.3.305

[8] Wik L, Kramer-Johansen J, Myklebust H, Sørebø H, Svensson L, Fellows B, et al Quality of cardiopulmonary resuscitation during out-of-hospital cardiac arrest. JAMA: the Journal of the American Medical Association. 2005;293(3):299–304. doi: 10.1001/jama.293.3.299 15657322 10.1001/jama.293.3.299

[9] Abella BS, Edelson DP, Kim S, Retzer E, Myklebust H, Barry AM, et al CPR quality improvement during in-hospital cardiac arrest using a real-time audiovisual feedback system. Resuscitation. 2007;73(1):54–61. doi: 10.1016/j.resuscitation.2006.10.027 17258853 10.1016/j.resuscitation.2006.10.027

[10] Kramer-Johansen J, Myklebust H, Wik L, Fellows B, Svensson L, Sørebø H, et al Quality of out-of-hospital cardiopulmonary resuscitation with real time automated feedback: a prospective interventional study. Resuscitation. 2006;71(3):283–292. doi: 10.1016/j.resuscitation.2006.05.011 17070980 10.1016/j.resuscitation.2006.05.011

[11] Yeung J, Meeks R, Edelson D, Gao F, Soar J, Perkins GD. The use of CPR feedback/prompt devices during training and CPR performance: a systematic review. Resuscitation. 2009;80(7):743–751. doi: 10.1016/j.resuscitation.2009.04.012 19477574 10.1016/j.resuscitation.2009.04.012

[12] Gruber J, Stumpf D, Zapletal B, Neuhold S, Fischer H. Real-time feedback systems in CPR. Trends in Anaesthesia and Critical Care. 2012;2(6):287–294. doi: 10.1016/j.tacc.2012.09.004

[13] Aase SO, Myklebust H. Compression depth estimation for CPR quality assessment using DSP on accelerometer signals. IEEE Transactions on Biomedical Engineering. 2002;49(3):263–268. doi: 10.1109/10.983461 11876291 10.1109/10.983461

[14] Ruiz de Gauna S, González-Otero DM, Ruiz J, Russell JK. Feedback on the Rate and Depth of Chest Compressions during Cardiopulmonary Resuscitation Using Only Accelerometers. PloS one. 2016;11(3):e0150139 doi: 10.1371/journal.pone.0150139 26930061 10.1371/journal.pone.0150139 PMC4773040

[15] Gohier F, Dellimore K, Scheffer C. Development of a real-time feedback algorithm for chest compression during CPR without assuming full chest decompression. Resuscitation. 2014;85(6):820–825. doi: 10.1016/j.resuscitation.2014.03.003 24632512 10.1016/j.resuscitation.2014.03.003

[16] González-Otero DM, Ruiz JM, Ruiz de Gauna S, Irusta U, Ayala U, Alonso E. A new method for feedback on the quality of chest compressions during cardiopulmonary resuscitation. BioMed research international. 2014;2014. 10.1155/2014/865967 PMC4163344 25243189

[17] Tomlinson AE, Nysaether J, Kramer-Johansen J, Steen P, Dorph E. Compression force-depth relationship during out-of-hospital cardiopulmonary resuscitation. Resuscitation. 2007;72(3):364–370. doi: 10.1016/j.resuscitation.2006.07.017 17141936 10.1016/j.resuscitation.2006.07.017

[18] Reynolds JC, Grunau BE, Rittenberger JC, Sawyer KN, Kurz MC, Callaway CW. Association Between Duration of Resuscitation and Favorable Outcome After Out-of-Hospital Cardiac ArrestClinical Perspective. Circulation. 2016;134(25):2084–2094. doi: 10.1161/CIRCULATIONAHA.116.023309 27760796 10.1161/CIRCULATIONAHA.116.023309 PMC5173423

[19] Silver A, Vadeboncoeur T, Venuti M, Tobin J, Smith G, Mullins M, et al Chest compression quality declines in the minutes preceding scene departure in out-of-hospital cardiac arrest. Resuscitation. 2013;84:S27 doi: 10.1016/j.resuscitation.2013.08.080

[20] Committee ECC, Subcommittees and Task Forces of the American Heart Association. 2005 American Heart Association guidelines for cardiopulmonary resuscitation and emergency cardiovascular care. Circulation. 2005;112(24 Suppl):IV1. 16314375 10.1161/CIRCULATIONAHA.105.166550

[21] Cho GC. Skin and soft tissue damage caused by use of feedback-sensor during chest compressions. Resuscitation. 2009;80(5):600 doi: 10.1016/j.resuscitation.2009.02.014 19362406 10.1016/j.resuscitation.2009.02.014

[22] Perkins GD, Kocierz L, Smith SC, McCulloch RA, Davies RP. Compression feedback devices over estimate chest compression depth when performed on a bed. Resuscitation. 2009;80(1):79–82. doi: 10.1016/j.resuscitation.2008.08.011 18952361 10.1016/j.resuscitation.2008.08.011

[23] Nishisaki A, Nysaether J, Sutton R, Maltese M, Niles D, Donoghue A, et al Effect of mattress deflection on CPR quality assessment for older children and adolescents. Resuscitation. 2009;80(5):540–545. doi: 10.1016/j.resuscitation.2009.02.006 19342150 10.1016/j.resuscitation.2009.02.006

[24] Ruiz de Gauna S, González-Otero DM, Ruiz J, Gutiérrez J, Russell JK. A Feasibility Study for Measuring Accurate Chest Compression Depth and Rate on Soft Surfaces Using Two Accelerometers and Spectral Analysis. BioMed research international. 2016;2016 doi: 10.1155/2016/6596040 27999808 10.1155/2016/6596040 PMC5143701

[25] Travers AH, Perkins GD, Berg RA, Castren M, Considine J, Escalante R, et al Part 3: Adult Basic Life Support and Automated External Defibrillation 2015 International Consensus on Cardiopulmonary Resuscitation and Emergency Cardiovascular Care Science With Treatment Recommendations. Circulation. 2015;132(16 suppl 1):S51–S83. doi: 10.1161/CIR.0000000000000272 26472859 10.1161/CIR.0000000000000272

